# Implementation of prehospital point-of-care ultrasound using a novel continuous feedback approach in a UK helicopter emergency medical service

**DOI:** 10.1186/s13049-025-01340-3

**Published:** 2025-02-04

**Authors:** Salman Naeem, Shadman Aziz, Thomas Hirst, Johannes Strobel, Jamin M. Mulvey, Ailidh Lang, Jankee Patel, Alexander Smith, Ka Jun Cheng, Michael Palmer, Jonas Schlautmann, Michael D. Christian, Daniel Nevin

**Affiliations:** 1 London’s Air Ambulance Charity, Royal London Hospital, London, UK; 2 Department of emergency medicine, William Harvey Hospital, East Kent Hospitals University Foundation Trust, Ashford, UK; 3 Department of Research, Audit, Innovation, and Development, East Anglian Air Ambulance, Norwich, UK; 4 Department of emergency medicine, Queen’s Hospital Romford, Havering and Redbridge University Hospitals NHS Trust, Barking, Romford, UK; 5 Department of Anaesthesia, Royal Free Hospital, London, UK; 6 Feuerwehr Hamburg EMS, Hamburg, Germany; 7 Department of Anaesthesiology, Queensland Children’s Hospital, QLD, Australia; 8 Department of Emergency Medicine, University College London Hospitals NHS Foundation Trust, London, UK; 9 Department of Emergency Medicine, London North West University Healthcare NHS Trust, London, UK; 10 Department of Critical Care, Guy’s and St Thomas’ NHS Foundation Trust, London, UK; 11 Department of Emergency Medicine, Maidstone and Tunbridge Wells NHS Trust, Tunbridge Wells, UK; 12 London Ambulance Service NHS Trust, London, UK; 13 Department of Acute Medicine, University Hospital Sussex, Brighton, UK; 14 Dept of Critical Care Medicine, University British Columbia, British Columbia, Canada; 15 Department of Anaesthesia, Royal London Hospital, Bart’s Health NHS Trust, London, UK

**Keywords:** PoCUS, Prehospital, HEMS, Trauma, Pneumothorax, Pericardial effusion, Shock

## Abstract

**Background:**

There has been increased use of prehospital point-of-care ultrasound (PoCUS) by helicopter emergency medical services (HEMS) in recent years. Lack of governance structure and evidence of benefit have been described as major barriers to its implementation. This paper describes a novel approach to implementation of prehospital PoCUS and clinical governance framework in a UK HEMS.

**Methods:**

A retrospective database review was undertaken at London’s Air Ambulance (LAA) from 1st September 2021 to 31st March 2023. All patients who had PoCUS examination were included. Scans were archived in a cloud-based server and reviewed weekly by expert clinicians. They were graded in adequacy, agreement between reviewer and clinician was recorded and fed back to the clinicians allowing continuous feedback learning. In-hospital diagnosis was sought for patients having the full Pump, Pleura and Pouring blood (PPPB) protocol. Cohen’s Kappa (ƙ) was calculated for inter-rater reliability. Sensitivity and specificity analysis was performed using 2 × 2 tables.

**Results:**

LAA attended 3,068 missions. Our reviewers identified 701 PoCUS scanning encounters and 628 were included in the final analysis. Clinicians performed 420 scans for pneumothorax, 308 for free fluid and 305 pericardial effusions respectively. Majority of the population were male (85%) who sustained traumatic (93.5%) thoracic injuries (65%). Paramedics performed 29% of the scans. Reviewers deemed 83% of the scans of adequate quality. Inter-rater reliability between clinicians and reviewers was 0.6 for pericardial effusion, 0.67 for pneumothorax and 0.71 for free fluid respectively. A full PPPB protocol was performed in 52 patients out of which 46 were included. The sensitivity and specificity of PPPB protocol for diagnosis life-threatening injuries was 0.5 and 0.9 respectively.

**Conclusion:**

Introduction of prehospital PoCUS in a HEM service utilizing high quality training, user-friendly workflow and image archiving system, robust governance framework and continuous feedback may be feasible allowing high quality ultrasound examinations. The bespoke PPPB protocol in prehospital may improve diagnosis of life-threatening injuries.

**Supplementary Information:**

The online version contains supplementary material available at 10.1186/s13049-025-01340-3.

## Introduction

Trauma is a leading cause of morbidity and mortality worldwide [1], necessitating timely and accurate assessment to guide appropriate interventions [2]. One critical aspect of prehospital trauma care is early recognition and management of shock [3], a state of inadequate tissue perfusion that can rapidly lead to organ dysfunction and death if left untreated [4]. Although haemorrhage is the leading cause of shock, critically injured patients can have other underlying causes of shock, i.e. obstructive, cardiogenic, and distributive [5]. They can also present with more than one cause for shock. Accurate and reliable diagnosis of shock in the prehospital setting is challenging, as time constraints and limitations in examination may hinder comprehensive evaluation [6].

Point-of-care ultrasound (PoCUS), the acquisition, interpretation and integration of sonographic imaging at a patient’s bedside by a non-radiologist and non-cardiologist treating clinician [7], has been widely used in hospitals for decades as a bedside tool for the diagnosis of life-threatening pathologies [8–10]. It allows real-time visualisation of anatomical structures and physiological changes, aiding in rapid diagnosis and management of shock [11]. Various protocols have been developed for systematic evaluation of patients in shock [12–14]. Extended-focused abdominal sonography in trauma (eFAST) is one such protocol recommended by the Advanced Trauma Life Support programme for the evaluation of injured patients [14].

In recent years due to technological improvements and increased portability of ultrasound devices, PoCUS has emerged as a significant innovation in prehospital care. It improves diagnostic yield and impacts time to intervention and definitive management [15]. However, challenges in training, governance and the potential to delay on-scene time remain limiting factors in its deployment in prehospital [16]. Many prehospital services, due to rotational workforce staff, have limited time to certify competency of clinicians in the use of PoCUS. This makes it challenging to utilise a lengthy and extensive formal sign-off approach for prehospital PoCUS. London’s Air Ambulance (LAA) employed a novel training approach to reintroduce prehospital PoCUS into service in September 2021 as an adjunct to clinical examination and guide time-sensitive interventions. This paper describes the implementation of the novel approach and its impact on clinicians’ management.

## Methods

### Study setting

LAA is a UK helicopter emergency medical service (HEMS) that provides prehospital advanced trauma care to a predominantly urban population of up to 12 million in a geographical area of approximately 2500 km^2^. At least one physician-paramedic team are available for dispatch 24 h a day by either helicopter or rapid response vehicle, depending on patient location, weather, and time of day. It is a primary major trauma response service and may occasionally encounter medical patients on ambulance crew requests. The service attends approximately 2000 patients per year, many of whom have injuries from penetrating trauma, accounting for approximately a third of its missions. This is followed by road traffic collisions and falls from height, which account for approximately 25% and 22% of missions respectively [17].

### Prehospital ultrasound at LAA

**Fig. 1:**
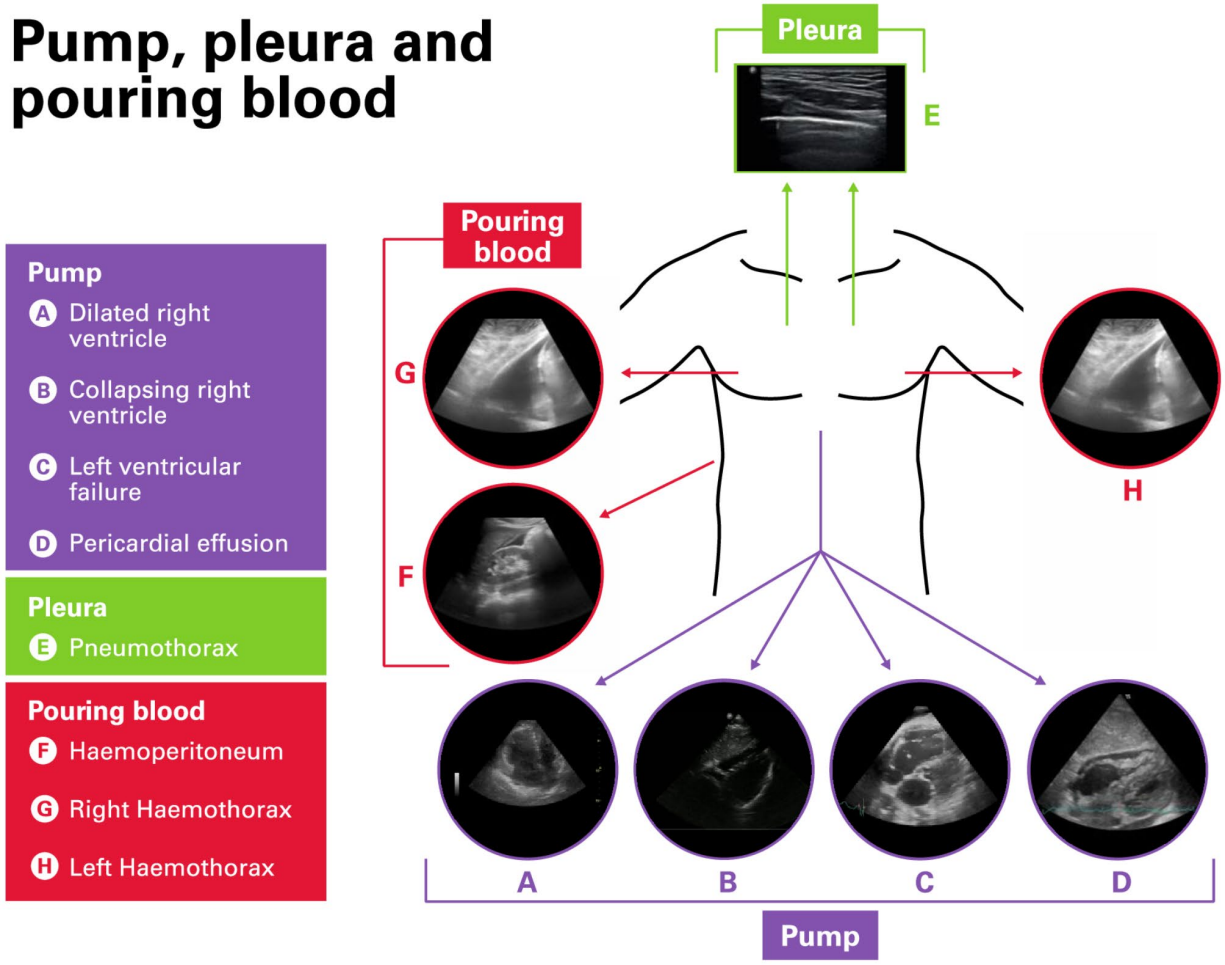
Ultrasound probe location for image acquisition of the “Pump, Pleura and Pouring Blood” Protocol. Legend: Components of “Pump, pleura and pouring blood” scanning protocol. The pump component investigates the heart using the subcostal 4 chamber window, for signs of obstructive shock (**A**: dilated right ventricle and **D**: pericardial effusion), low preload (**B**: collapsing right ventricle), cardiogenic shock (**C**: poorly contracting left ventricle). The pleura component includes scanning both hemithoraces from 2nd to 4th intercostal space in the mid-clavicular line scanning for absence of lung sliding (**E**: pneumothorax). The pouring blood component investigates for occult bleeding the torso (**F**: right upper quadrant for free fluid in the peritoneum, **G** & **H** for pleura effusion in both hemithoraces)

After previously being trialled in 2012, PoCUS was re-introduced into LAA in September 2021 to assist clinicians in diagnosing of the cause of shock in patients. In view of the unique population of severely injured patients encountered by the service; the ‘Pump, pleura and pouring blood’ (PPPB) protocol was developed by LAA to answer binary questions relevant to critically injured trauma patients without significantly delaying prehospital scene or transport time. This bespoke, truncated PoCUS protocol can be performed by the HEMS clinicians (either physicians or paramedics) to rapidly gather relevant information, prioritise, and guide resuscitative efforts in time-critical trauma patients. The elements of the protocol and the location of image acquisition is shown in Fig. 1 with description of the protocol (Additional file 1).

The findings of the protocol are used to guide resuscitative interventions in shocked trauma patients (Table 1).

**Table 1:** Resuscitative interventions utilised at London’s Air Ambulance for shocked trauma patients

Pathology	Intervention
Hypovolaemia	Volume replacement with blood products
Tension pneumothorax	Surgical thoracostomy
Cardiac tamponade	Preparation of the potential need for resuscitative thoracotomy (RT).
Subdiaphragmatic haemorrhage	Aortic occlusion with resuscitative endovascular balloon occlusion of the aorta (REBOA) or RT
Massive haemothorax	Volume replacement or RT if indicated

All LAA clinicians are trained to perform the PPPB protocol, which allows any member of the HEMS team to perform the examination in parallel with the resuscitative efforts of other team members. During patient handover in hospital, only positive findings are relayed to the trauma team leader to expedite treatment of critically injured patients, and to avoid introduction of any bias.

### Training and competency

All clinicians follow a standardised training program to ensure uniformity of training and competence during their training period. A bespoke teaching and training programme was developed for prehospital PoCUS at LAA. All clinicians undertake a two-hour online modular training package which included basic physics, image acquisition and interpretation of physiological and pathological ultrasound clips for PPPB protocol. This is followed by a two-hour hands-on workshop with the ultrasound lead, detailing knobology and practice of focused and limited image acquisition [1]. Clinicians spend a 6-week period under intense scrutiny and supervision (initial training period for HEMS). All PoCUS scans are reviewed, and direct feedback is delivered in written format back to the scanning clinician. Due to the transient nature of rotating clinicians (6 months doctor secondment, 12 months paramedic secondments), it was recognised that competency frameworks and sign-off processes for in-hospital ultrasound faculties as used by the Royal College of Emergency Medicine [19] would be impractical. Using this approach of continuous performance feedback [20] oversight and governance meant a move away from formal sign-off to a process of ongoing mentorship for our clinicians hence, there is no formal sign-off process in the service.

### Ultrasound device and workflow

**Fig. 2:**
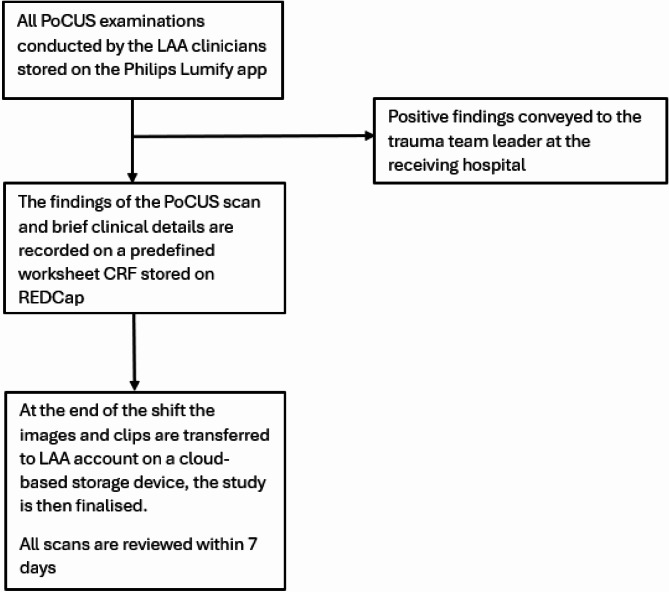
LAA PoCUS workflow. Legend: A flowsheet showing the workflow of point-of-care ultrasound (PoCUS) examinations performed at London’s Air Ambulance (LAA). Abbreviations: PoCUS: Point-of-care ultrasound, LAA: London’s Air Ambulance CRF: Clinical Review Form

Several contemporary handheld devices were reviewed and after careful consideration the Philips Lumify handheld device was selected for prehospital PoCUS at LAA. It was chosen due to its image quality, weight, size, interchangeability of the probe, user-friendly interface and lack of battery in the probe which reduced the equipment needed to be carried. The device is carried in a bespoke carrier pouch (Additional file 2). The Phillips Lumify device uploads data to cloud-based software and is fully GDPR compliant. In addition, clinicians capture only the Computer Aided Dispatch code provided by the control room to tag the images. The device automatically date and time stamps the images. The images are thus anonymised unless these three pieces of information are correlated with the information on the encrypted service database to correlate the images to a particular job or patient. The workflow for LAA prehospital PoCUS is summarised below in Fig. 2.

A separate documentation clinical review form (CRF) template was created on REDCap secure data environment (V.8.1.20. Vanderbilt, Tennessee, USA (Additional file 3) which is filled by clinicians performing the scans for all patient encounters.

### Clinical governance

Recording of the PoCUS examination is mandatory at LAA to allow for retrospective review. Images and clips are archived on a secure cloud-based system at the end of the shift. These scans are reviewed by a prehospital PoCUS specialist on a weekly basis and feedback is provided for each scan on image quality (including depth, gain and orientation), agreement between the reviewer and the scanning clinician and feedback on image interpretation. The reviewers use a standardized template (Additional file 4) to provide feedback on each scan which is stored on REDCap. Twice a year, archived images, reviewer reports and feedback are randomly reviewed by an external PoCUS expert. These processes allow for quality assurance and institutional learning around prehospital PoCUS.

### Data collection

A retrospective database review was conducted on a consecutive sample of all patients attended by LAA from introduction of prehospital PoCUS in September 2021 to March 2023. The project was registered as a service evaluation with the clinical effectiveness unit at Barts Health NHS Trust (audit registration number 12927). Data was collected from the previously utilised LAA database (OnBase version EP1, Hyland Software, Ohio, USA) between September 2021 and July 2022 and the current service database on Microsoft PowerApps (V 3.22055.9) between July 2022 and March 2023. All patients attended by LAA who had PoCUS examination and corresponding quality assurance process completed by 31st of March 2023 were included in the review. Patients who had the full PPPB protocol were followed up and their in-hospital diagnosis, injuries and mortality were included. In-hospital data were extracted by trained abstractors from their respective electronic patient records at each hospital site. Study data were collected, anonymised and stored in the REDCap secure data environment (V.8.1.20. Vanderbilt, Tennessee, USA).

Variables collected included: timings of the prehospital incident, age, sex, mechanism of injury, suspected injuries, PoCUS windows obtained, prehospital PoCUS findings, issues with prehospital scanning, impact of PoCUS on patient management, reviewer data on adequacy and agreement between performer and reviewer, ‘code red’ (defined as any trauma patient with a systolic blood pressure < 90 mmHg with suspected or confirmed haemorrhage and no response to fluids) declared at any time, in-hospital findings, patient outcome. Data abstractors used the following sources to establish the patients’ definitive injury burden: clinical examination, consultant radiologist reports (CT and chest radiography), in-hospital ultrasound reports (both PoCUS and formal sonography) and intra-operative findings. Any pneumothorax, pericardial effusion and free fluid in the chest or abdomen was considered life-threatening injury for analysis of sensitivity and specificity of PPPB protocol where it was performed in its entirety on patients.

The primary outcome was the degree of inter-rater reliability between the expert reviewers and the HEMS clinicians performing the scan. Secondary outcomes included the median scene times of the missions, impact of PoCUS on patient management and the accuracy of the PPPB in diagnosing life-threatening injuries (i.e., pericardial effusion, pneumothorax, and free fluid).

### Data analysis

Data were exported to Microsoft SPSS 29.0 (SPSS Statistics, IBM, Armonk, NY, USA) for analysis. Sample characteristics were described using percentage for categorical variables and mean (+/- Standard deviation (SD)) for continuous variables. Cohen’s kappa was calculated for inter-rater reliability between reviewers and clinicians for each modality. For a subset of patients who had the whole PPPB protocol 2 × 2 table was calculated to report its sensitivity and specificity. Non-parametric variables were compared using Mann-Whitney U test.

## Results

**Fig. 3:**
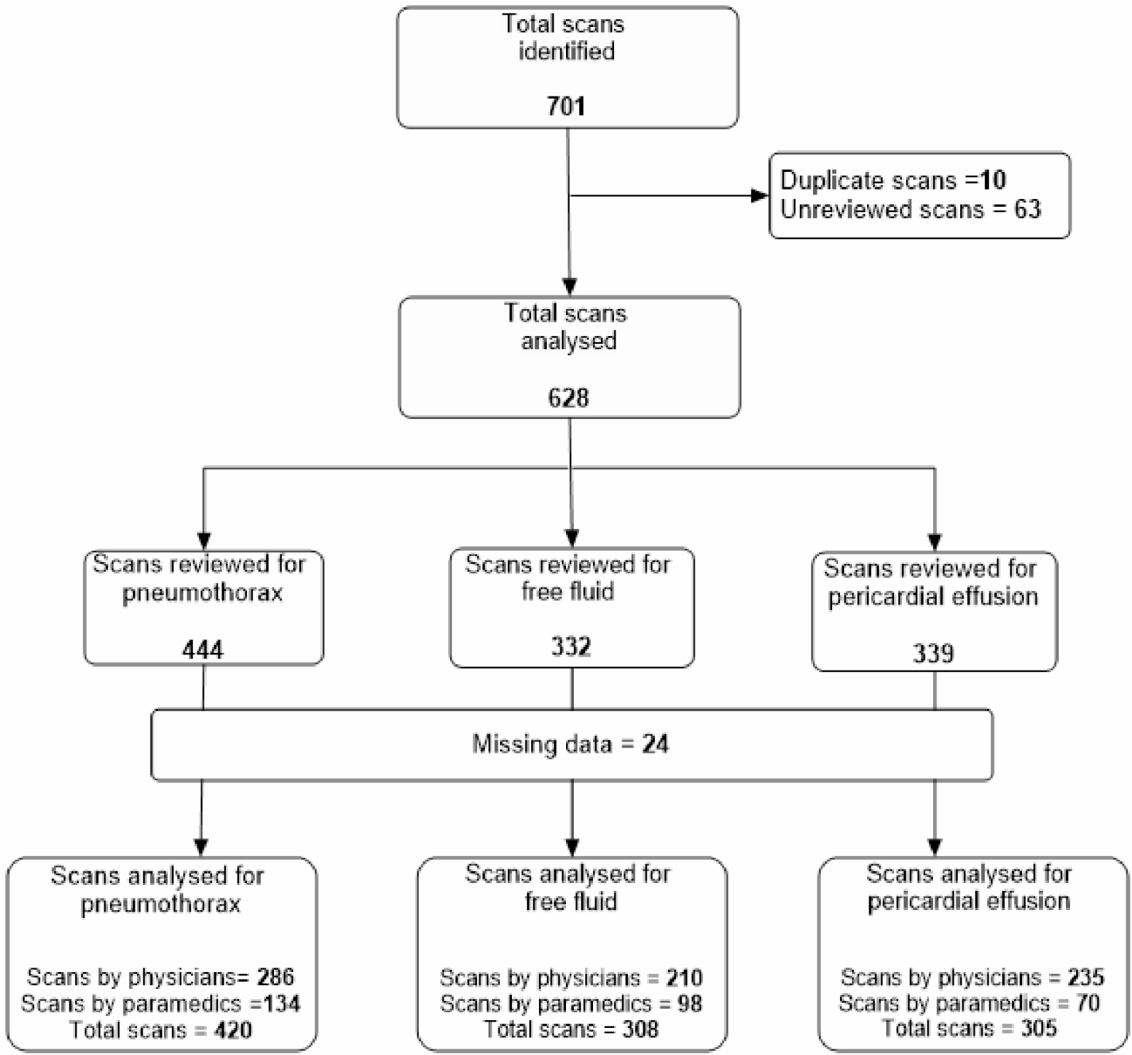
Participant flowchart for the study. Legend: Flowchart showing screening, inclusion and distribution of prehospital ultrasound scans in the review

LAA attended 3,068 missions during the study period. Our reviewers identified 701 PoCUS scanning encounters archived. After removing duplicates and unreviewed scans by 31st of March 2023, 628 were analysed as described in Fig. 3.

Basic demographics are detailed in Table 2. PoCUS was used in 23% of the total missions attending by LAA during the study. Median scene time for the missions in which PoCUS was performed was 22.50 (IQR 14–37) minutes and it was 21 (IQR 12–33) minutes where it was not performed. It was significantly higher in missions where PoCUS was performed.

**Table 2:** Demographics of population included in the service evaluation

Demographics	*n* (%)
Sex	
Male	536 (85%)
Female	92 (15%)
Age category	
Adult	410 (89%)
Paediatric	50 (11%)
Pathology	
Trauma	587 (93.5%)
Medical	41 (6.5%)
Suspected injuries	
Thorax	260 (65.5%)
Abdomen	113 (28.5%)
Traumatic cardiac arrest	24 (6%)
Operator specialty	
Doctor	480 (71%)
Paramedic	180 (29%)

Data expressed as n= (%) unless otherwise stated

Reviewers deemed 83% of the scans of adequate quality with low inter-rater reliability between the clinicians and the reviewers. Inter-rater reliability measured as Cohen’s Kappa for paramedics and physicians is given in Table 3.

**Table 3:** Inter-rater reliability (Cohen’s Kappa) between the reviewers and paramedics, physicians and combined population for pericardial effusion, pneumothorax and free fluid

Cohen’s Kappa	Physicians	Paramedics	Combined
Pericardial effusion	0.70	0.14	0.60
Pneumothorax	0.71	0.92	0.67
Free fluid	0.75	0.59	0.71

Inter-rater reliability represented by Cohen’s Kappa co-efficient

Clinicians reported the impact of their scans on their management in 533 encounters where it allowed confirmation of diagnosis, rule-out diagnoses, supported clinical plan and added no value in 57%,26%,14% and 2.4% respectively. Issues with ultrasound machine, patient, environment, and operator were reported in 13%, 16%, 14% and 3% of encounters respectively.

A full PPPB protocol was performed in 52 patients out of which 46 were included in the final analysis. Code red was declared in nine patients. The sensitivity and specificity of PPPB protocol for diagnosis life-threatening injuries i.e. pericardial effusion, pneumothorax or free fluid using in-hospital CT scan or intra-operative findings as gold standard are given in Table 4.

**Table 4:** Sensitivity and specificity of the PPPB protocol for detecting any major traumatic pathology (pericardial effusion, pneumothorax, or free fluid)

	Sensitivity (95% CI)	Specificity (95% CI)
All patients	0.56 (0.37–0.73)	0.90 (0.71–0.98)
Code red patients	0.75 (0.46–0.96)	0

## Discussion

Prehospital PoCUS is being used in HEMS across Europe [20] however, lack of governance and training standards, evidence of benefit and delay in patient management have been described as barriers to its implementation by Naeem et al. [18]. In this study we describe a novel approach to introduction of prehospital PoCUS at LAA along with and audit of the data.

This initiative demonstrates that prehospital PoCUS is not only feasible but also reliable when it is deployed with targeted training, practised within a limited scope, and instituted with high-quality governance and continuous feedback [20]. Scans conducted by our clinicians, including physicians and paramedics, were mostly of adequate quality (83%) which are similar to the results reported by Stroney et al. on prehospital eFAST [22]. Furthermore, our results are comparable to ones achieved by credentialed clinicians performing prehospital PoCUS [22–24]. There was substantial agreement between the performers and reviewers for diagnosis of pericardial effusion, pneumothorax and free fluid in chest and abdomen which shows that with limited training and continuous feedback the interpretation of prehospital PoCUS findings were of high-quality achieving results similar to clinicians who have undergone formal accreditation [22, 23].

Diagnosis of injuries in prehospital environment can be challenging. Mechanism of injury and physical exam have limitations and experienced HEMS clinicians are only moderately accurate [6, 26]. Prehospital ultrasound has been shown to have improved accuracy in diagnosis of underlying pathology and reduce time to intervention [22, 27]. This study shows substantial inter-rater reliability of the scans performed possibly improving the diagnostic accuracy of the clinicians. However, due poor availability of hospital data, we did not perform sensitivity and specificity analysis. Hence, we are unable to postulate about the diagnostic accuracy of PoCUS in our cohort.

Diagnosis of shock and or life-threatening injuries is paramount in prehospital care. FAST has been used in prehospital for diagnosis of occult bleeding [22–25, 27] but it does not investigate the heart in terms of its function and filling [24]. Hence, LAA introduced a bespoke scanning protocol to its service i.e. PPPB, which allows the diagnosis of shock and assessment of the heart. In our study, the sensitivity of PPPB for picking up life-threatening injuries was 56% in all trauma patients, which increased to 75% in patients with life-threatening haemorrhages. This is similar to the performance of prehospital eFAST in picking up underlying injuries [28]. However, our protocol allows comprehensive evaluation of causes of shock within limited time with comparable results. The lower sensitivity of PPPB in all trauma patients could be either due to missing insignificant injuries or that the clinicians performed the scans in the hyperacute, acute phase of the injury. This is evident by increased sensitivity in patients with haemodynamic compromise. The main aim of this study was not to validate the PPPB protocol and along with limited numbers the result of the sensitivity and specificity analysis should be interpreted with caution.

One of the postulated pitfalls of prehospital PoCUS is that it may prolong the on-scene time and delay definitive care [16]. In our study the on-scene time was significantly higher in missions when PoCUS was performed. It is difficult to associate the delay to PoCUS due to small sample size and inability to control for confounders i.e. day versus night jobs, severity of injuries and other interventions, complexity of scene and delays due to other environmental and scene factors. PoCUS in our service is often performed in the ambulance enroute to the hospital hence it is unlikely that it would have caused delay in on-scene management. Onotera et al. demonstrated no significant delays due to prehospital ultrasound [29]. Another barrier to implementation of prehospital PoCUS is evidence of its benefit [16]. Current literature reports evidence for change in management, disposition and mode of transport [27–30]. Clinicians found prehospital PoCUS useful in 97% of the patients where it allowed to confirm or rule-out diagnosis and supported their current plan of treatment. This study did not investigate effects of prehospital PoCUS on disposition as almost all trauma patients attended by LAA are transported to the local major trauma centres.

In UK, there is no specific guidance for prehospital PoCUS from the Faculty of Pre-hospital Care. However, the Royal College of Radiologists in collaboration with British Medical Ultrasound Society have published recommendations for practice of PoCUS which highlights key principles for establishing and maintaining a PoCUS program in an organisation (governance, image archiving, education and training, image review and audit) [31]. The implementation of prehospital PoCUS at LAA followed the key principles, however, in our model we did not have any summative assessments for our clinicians performing prehospital PoCUS. The continuous feedback model, where all scans were reviewed, and feedback was given to the clinicians allowed high quality scans in the prehospital environment with substantial inter-rater reliability in diagnosing major pathologies. This contrasts with the formal sign-off processes by various organisations for in-hospital [19, 32].

### Limitations

This study has several limitations. First, the study has used retrospective data which may predispose this to information bias. Second, the generalisability of this study to other prehospital settings is limited as it only included patients attended by a single HEMS, who are predominantly dispatched to traumatic incidents in an urban environment. Third, we were not able to acquire follow up hospital data for all patients, hence we were unable to calculate sensitivity and specificity of prehospital ultrasound for all conditions. This was due to lack of data sharing agreements between the hospitals and LAA. Fourth, this study was not designed to validate the PPPB protocol, hence the sensitivity and specificity results should be interpreted with caution.

## Conclusion

In this retrospective review, we have described the feasibility of introducing of a high-quality prehospital PoCUS in a HEM service utilizing bespoke training, user-friendly workflow and image archiving system, robust governance framework and continuous feedback without having the need for length sign-off process for its clinicians. The bespoke PPPB protocol in prehospital may improve diagnosis of life-threatening injuries. This model may be used to establish PoCUS in other HEM services.

## Electronic supplementary material

Below is the link to the electronic supplementary material.

Supplementary Material 1

Supplementary Material 2

Supplementary Material 3

Supplementary Material 4

## Data Availability

No datasets were generated or analysed during the current study.

## Abbreviations

CRF: Clinical Review Form
E-FAST: Extended focused assessment with sonography for trauma
GDPR: General Data Protection Regulation
HEMS: Helicopter Emergency Medicine Service
IQR: Inter-quartile range
LAA: London’s air ambulance
PoCUS: Point-of-care ultrasound
PPPB: Pump, pleura and pouring blood
SD: Standard deviation
UK: United Kingdom
