# Supplementary material for: Implementation of prehospital point-of-care ultrasound using a novel continuous feedback approach in a UK helicopter emergency medical service
Source: Scand J Trauma Resusc Emerg Med. 2025 Feb 4;33:21. doi: 10.1186/s13049-025-01340-3 (PMC11796228; doi:10.1186/s13049-025-01340-3)
Supplement: Supplementary file 1 — Supplementary Material 1 [file 13049_2025_1340_MOESM1_ESM.docx]

**Additional file: Table 1:**

“Pump, Pleura and Pouring Blood” PoCUS protocol at London’s Air Ambulance

| **Pump**  A single subxiphoid cardiac window is obtained to answer the following binary question:   - Is there evidence of a pericardial effusion?   This view is also used to obtain an impression of the following:   - Is the heart empty? - Are the ventricles dilated or contracting poorly? |
| --- |
| **Pleura**  Depending on patient position, a two-point bilateral examination of the highest point of the chest wall is obtained to look for the presence or absence of lung sliding to answer:   - Is there evidence of a pneumothorax? |
| **Pouring blood**  Two specific examinations are used to identify torso haemorrhage. A bilateral examination of the most basal/dependent aspects of the chest (depending on patient position) to evaluate for the presence of fluid in the hemithorax; followed by a single examination of the right upper quadrant abdominal scan to evaluate for free fluid. This is to answer the following questions:   - Is there evidence of a haemothorax? - Is there evidence of a haemo-peritoneum? |
