# Supplementary material for: Implementation of prehospital point-of-care ultrasound using a novel continuous feedback approach in a UK helicopter emergency medical service
Source: Scand J Trauma Resusc Emerg Med. 2025 Feb 4;33:21. doi: 10.1186/s13049-025-01340-3 (PMC11796228; doi:10.1186/s13049-025-01340-3)
Supplement: Supplementary file 2 — Supplementary Material 2 [file 13049_2025_1340_MOESM2_ESM.pdf]

POCUS Audit Form

Please complete the survey below.

Thank you!

Review date	__________________________
Reviewer	◯ Dan Nevin<br>◯ Matt Mak<br>◯ Behnaz Mahmoodian<br>◯ Richard Muswell<br>◯ Adrian Wong<br>◯ Salman Naeem<br>◯ Other
Other reviewer name:	__________________________
CAD number	__________________________
Scan date	__________________________
PoCUS images	◯ Adequate<br>◯ Poor
Pump	◯ Agree with findings<br>◯ Disagree with findings<br>◯ Image not captured
Details of why you disagree with the 'pump' interpretation	__________________________
Pleura	◯ Agree with findings<br>◯ Disagree with findings<br>◯ Image not captured
Details of why you disagree with the 'pleura' interpretation	__________________________
Pouring blood	◯ Agree with findings<br>◯ Disagree with findings<br>◯ Image not captured
Details of why you disagree with the 'pouring blood' interpretation	__________________________
Appropriate depth	◯ Yes<br>◯ No
Appropriate gain	◯ Yes<br>◯ No

Appropriate probe orientation	◯ Yes<br>◯ No
Appropriate window obtained	◯ Yes<br>◯ No
Other image problem/comment	__________________________________
General Comments/Feedback	__________________________________
