# Supplementary material for: Implementation of prehospital point-of-care ultrasound using a novel continuous feedback approach in a UK helicopter emergency medical service
Source: Scand J Trauma Resusc Emerg Med. 2025 Feb 4;33:21. doi: 10.1186/s13049-025-01340-3 (PMC11796228; doi:10.1186/s13049-025-01340-3)
Supplement: Supplementary file 3 — Supplementary Material 3 [file 13049_2025_1340_MOESM3_ESM.pdf]

POCUS CRF

Please complete the survey below.

Thank you!

Incident Date	__________________________________
CAD number	__________________________________
Adult or Child?	◯ Adult<br>◯ Child
Sex	◯ Male<br>◯ Female
Operator Name	__________________________________
Indication	◯ Trauma<br>◯ Medical (cardiac arrest)
Suspected Injuries	☐ Chest injuries<br>☐ Abdominal injuries<br>☐ Traumatic Cardiac arrest
PoCUS Images	◯ Poor<br>◯ Adequate

Pump

Pericardial fluid	◯ Yes<br>◯ No<br>◯ Not done<br>◯ Unable to assess
Right ventricle visually	◯ Collapsed<br>◯ Not dilated<br>◯ Dilated<br>◯ Not done<br>◯ Unable to assess
Left ventricle filling	◯ Well filled<br>◯ Poorly filled<br>◯ Not Done<br>◯ Unable to assess
Left ventricle contractility visually	◯ Good<br>◯ Poor<br>◯ Standstill<br>◯ Not done<br>◯ Unable to assess

Pleura

Right chest	◯ Pneumothorax<br>◯ Normal<br>◯ Not done<br>◯ Unable to assess
Left chest	◯ Pneumothorax<br>◯ Normal<br>◯ Not done<br>◯ Unable to assess

Pouring blood

Right haemothorax	◯ Yes<br>◯ No<br>◯ Not done<br>◯ Unable to assess
Left haemothorax	◯ Yes<br>◯ No<br>◯ Not done<br>◯ Unable to assess
Haemoperitoneum	◯ Yes<br>◯ No<br>◯ Not done<br>◯ Unable to assess

Problems encountered with PoCUS

Issue(s) encountered with the US machine	◯ Yes<br>◯ No
Issue(s) with	☐ Probe attachment or working<br>☐ Tablet power<br>☐ App functioning<br>☐ US controls in app<br>☐ Image acquisition<br>☐ Image/ video saving<br>☐ Other<br>(Select all that apply)
Issue(s) encountered with patient	◯ Yes<br>◯ No
Issue(s) with...	☐ Patient refusal<br>☐ Obese<br>☐ Frail<br>☐ Distorted anatomy<br>☐ Other<br>(Select all that apply)
Issue(s) encountered due to environment	◯ Yes<br>◯ No

Issue(s) with....	☐ Ambient light<br>☐ Limited access to patient<br>☐ Limited time<br>☐ Other<br>(Select all that apply)
Issue(s) encountered due to operator	◯ Yes<br>◯ No
Issue(s) with....	☐ Operator knowledge<br>☐ Operator confidence<br>☐ Image interpretation<br>☐ Other<br>(Select all that apply)
Clinical Context and Comments:	____<br>(Please provide a brief (one line e.g. "Male stabbed in right chest") description or any other comments relevant for the reviewers.)
How did the use of PoCUS add to the clinical interaction?<br>(Select all relevant options)	☐ Confirm diagnosis<br>☐ Rule out diagnosis<br>☐ Unexpected diagnosis<br>☐ Support proposed clinical plan or management strategy<br>☐ Alter proposed clinical plan or management strategy<br>☐ No value add
