# Supplementary material for: Implementation of prehospital point-of-care ultrasound using a novel continuous feedback approach in a UK helicopter emergency medical service
Source: Scand J Trauma Resusc Emerg Med. 2025 Feb 4;33:21. doi: 10.1186/s13049-025-01340-3 (PMC11796228; doi:10.1186/s13049-025-01340-3)
Supplement: Supplementary file 4 — Supplementary Material 4 [file 13049_2025_1340_MOESM4_ESM.docx]

Additional file 2


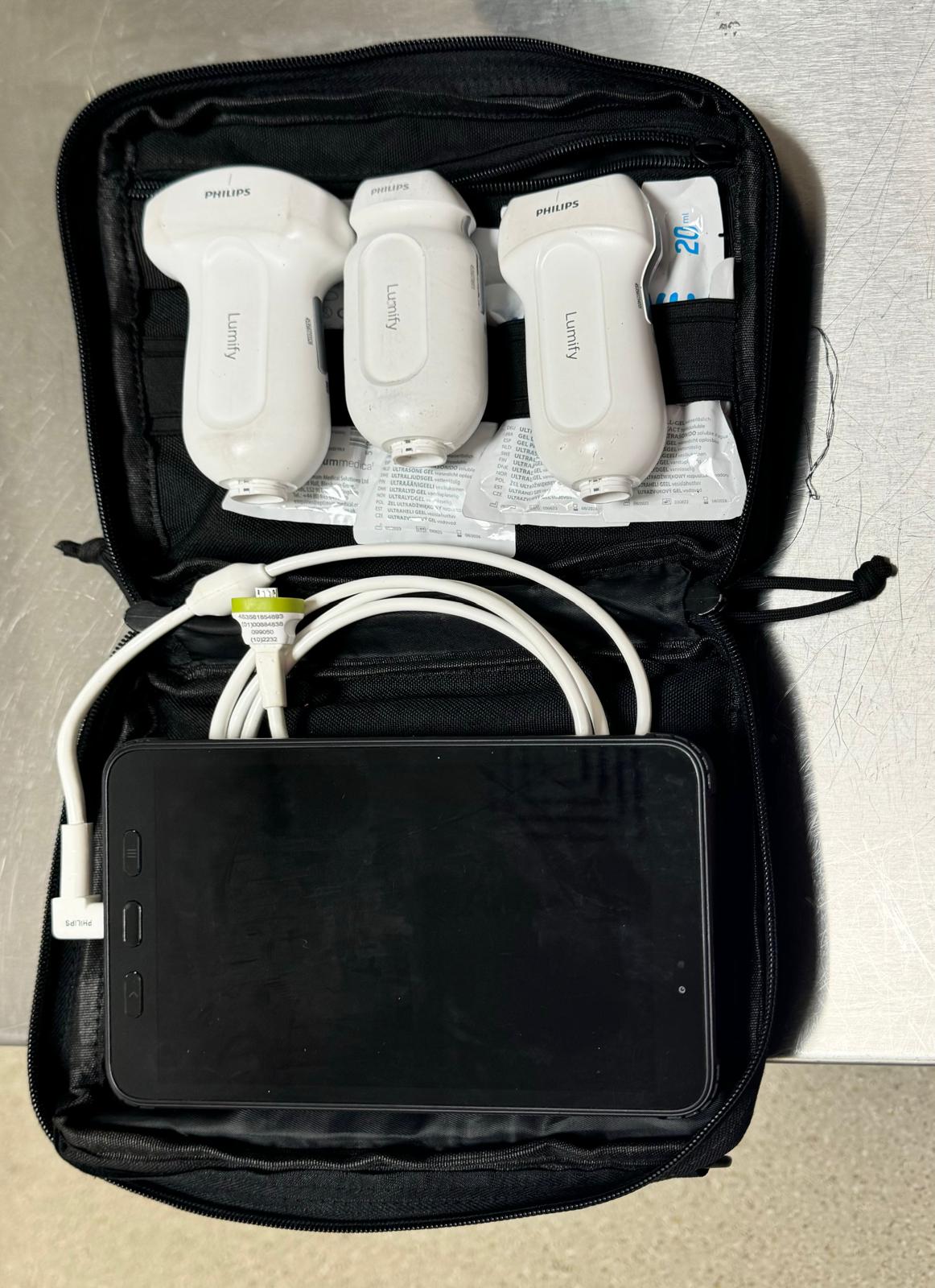


Figure 2: The PoCUS carrier pouch with I-pad, ultrasound cable, Philips Lumify linear, curvilinear and phased-array probes and ultrasound gel sachets.
